# Hydraulic path length as a determinant of xylem conduit size at the stem base, regardless of cambial age

**DOI:** 10.1093/treephys/tpaf127

**Published:** 2025-10-14

**Authors:** Giovanni Bicego, Mirko Cocco, Carlo Urbinati, Tommaso Anfodillo

**Affiliations:** Dipartimento Territorio e Sistemi Agro-Forestali, Università degli Studi di Padova, Viale dell’Università 16, 35020 Legnaro PD, Italy; Dipartimento Territorio e Sistemi Agro-Forestali, Università degli Studi di Padova, Viale dell’Università 16, 35020 Legnaro PD, Italy; Forest Ecosystems Unit – Department of Crop, Food and Environmental Sciences, Università Politecnica delle Marche, Via Brecce Bianche 10, 60131 Ancona AN, Italy; Dipartimento Territorio e Sistemi Agro-Forestali, Università degli Studi di Padova, Viale dell’Università 16, 35020 Legnaro PD, Italy

**Keywords:** angiosperms, conduit widening, crown structure, hydraulic sectoriality

## Abstract

Xylem properties, such as wood density and conduit diameter, are linked to crown shape and size. Suppressed trees with smaller crowns tend to have denser wood and narrower conduits at the stem base, whereas dominant trees exhibit wider conduits and lower wood density. Given the tip-to-base widening of xylem conduits—an adaptation to counteract increasing hydraulic resistance with growth—we hypothesize that hydraulic path length (i.e., the distance from leaves to the stem base) is the primary driver of conduit size, independent of cambial age. To test this, we leveraged a phenomenon in managed forests: partial harvesting reduces stand density, triggering epicormic shoot formation along the stems of uncut (standing) trees. This downward shift in leaf distribution shortens the average hydraulic path length, allowing us to assess its influence on conduit formation in the standing trees. If conduit size is governed by hydraulic path length, newly formed tree rings should contain narrower conduits following epicormic shoot sprouting, despite the older cambial age. We analysed wood samples from nine broadleaved trees across four species (*Acer opalus obtusatum* (Waldst. & Kit. Ex Willd.) Gams, *Ostrya carpinifolia* Scop., *Carpinus betulus* L., *Sorbus aria* (L.) Crantz.), comparing the median conduit area in three to four annual rings before and after the harvesting of neighbouring trees. In trees with epicormic shoots, conduit size decreased by a factor ranging from 0.93 to 0.56 (*P* < 0.01). Conversely, the two trees without epicormic shoots exhibited no significant changes in conduit size. Our findings indicate that conduit size at the stem base is determined by hydraulic path length, rather than by cambial age. This suggests that newly formed leaves regulate the vascular conduits supplying them, leading to a hydraulic network structured by multiple, axially sectored pathways.

## Introduction

Among living organisms, trees exhibit an unparalleled range in size during their growth (Enquist 2002). This exceptional change in dimensions also involves an adjustment of structures that should allow maintenance of the overall functionality of the organism. One of the most significant scalings of plant attributes with size concerns the anatomical variation of the water transport network.

Analysing anatomical variations across species and conditions helps to understand the significance of specific xylem traits and patterns in determining individual fitness. For instance, the fact that in taller trees, at the stem base, xylem conduits are consistently wider than those in corresponding smaller individuals in the same site demonstrates the adaptive importance of tip-to-base conduit widening (Anfodillo et al. 2006, Olson and Rosell 2013, Rosell et al. 2017, Koçillari et al. 2021). The basipetal increase in conduit size helps maintain a relatively constant overall hydraulic resistance despite the increase in hydraulic path length. In other words, widening of xylem conduits is an effective feature to supply distal leaves with the same water flow, regardless of the absolute root-to-leaf distance (Becker et al. 2003). The rate of conduit widening from leaves to stem base was demonstrated to be rather stable among plants of vastly different habits and habitats (Olson and Rosell 2013, Olson et al. 2018, 2020). Conduit size increases with distance from tree top following approximately a power law with an exponent of about 0.2 along branches and stem (Anfodillo et al. 2013). Variants adopting the anatomical structure with ‘optimal’ widening are favoured over those with ‘too low’ and those with ‘too high’ widening. The former have lower fitness because, with growth, hydraulic resistance significantly increases, consequently reducing water flow to the leaves, thereby decreasing leaf productivity and growth (Scoffoni et al. 2016). The latter might be less fitted because having conduits ‘too wide’ at the base could increase the probability of embolism, considering the relationship between tangential conduit diameter and cavitation (Rosner et al. 2016, Rodriguez-Zaccaro et al. 2019) leading to reduced survivorship. Although the relationship between xylem conduit diameter and vulnerability remains debated (Lens et al. 2022), the greater susceptibility of wider conduits to cavitation would find empirical support in the observations that, on average, tall plants seem to be more sensitive to critical water conditions (Nepstad et al. 2007, Anfodillo and Olson 2021, Olson et al. 2023, Zambonini et al. 2024) even in ecosystems not usually affected by water deficit, such as tropical regions (Bennett et al. 2015).

The universality of the tip-to-base widening is also suggested by very diverse subject literature. For example, in wood science there is evidence that the anatomical properties of wood in the stem change in relation to crown structure, with denser wood (i.e., average narrower conduits) in stems of overtopped trees (Krajnc et al. 2019) and that wood density is positively correlated to crown depth (Iida et al. 2012). These findings can be explained because overtopped trees are, in general, smaller and have short branches compared with dominant trees, and the overall shorter water path length is associated with narrower conduits at the stem base.

Within the same line of reasoning the increase of conduit size radially at a given tree height (i.e., from the pit to the outermost rings) can be explained simply because the outermost rings were formed when the tree was taller (and older).

For the same reason the correlation is observed between conduit size and size of the organ (branch, stem or root) in poplar trees (Jacobsen et al. 2018), because wider conduits are found in wider organs that are further from the tree top.

We are very aware, however, that the correlation between age and wood structure has been extensively proposed in a substantial body of literature (Lenz et al. 2010, Schulte 2012, Jacobsen et al. 2018, Rodriguez-Zaccaro et al. 2019). According to these empirical observations, the older the cambium, the wider the conduits are that it produces, although, to our knowledge, no solid theory has been developed to explain the causal mechanism linking cambial age to conduit size.

**Figure 1:**
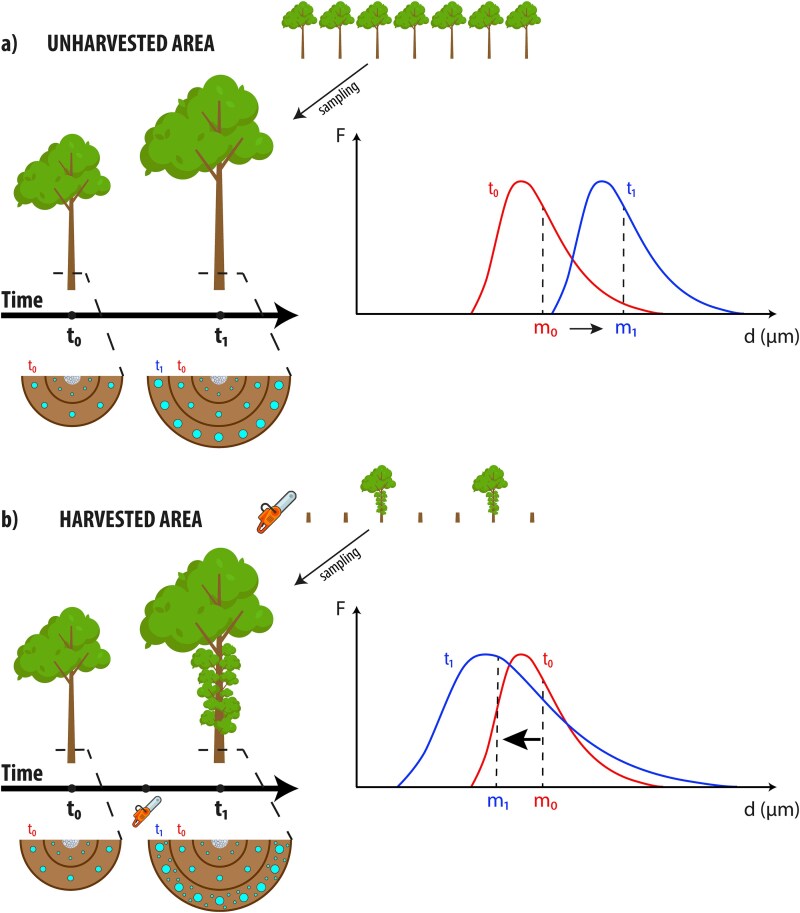
Predicted variations of conduit distribution (i.e., wood anatomy) at the stem base due to the inescapable tip-to-base conduit widening. (a) With ontogenetic growth (from *t*_0_ to *t*_1_) the median of conduit diameter (*m*) increases (from *m*_0_ to *m*_1_) as trees become taller due to the average longer length of the hydraulic path. (b) However, if some events lead to crown shape variation (i.e., a sudden exposure to full light of mature trees with the concomitant sprouting of epicormic shoots in the lowest portion of the stem), then the anatomy of the new xylem tissue should change in a predictable way. In the rings formed after harvesting, we observe the formation of both narrower conduits, likely serving the newly developed lower leaves, and wider conduits, connected to the upper canopy. The distribution of conduit size after harvesting (*m*_1_) tends to shift towards narrower conduits compared with previous rings (*m*_0_), in spite of an increase in cambial age. This provides a robust experimental foundation to prove that hydraulic path length, rather than cambial age, is the primary causal factor determining conduit diameter.

We planned a simple manipulation experiment to demonstrate that the hydraulic path length is the primary driver of conduit anatomy at the stem base, thus falsifying a possible causal link to age-size. We conducted our experiment in Apennine forests managed as coppice-with-standards, a silvicultural system commonly employed in Southern Europe. After harvesting, only ~100 trees (standards) per hectare were left standing. The increased availability of light and other resources often induces the sprouting of a large number of epicormic shoots along the stem of uncut (standing) trees (Figure 1b). We sampled the standing trees and measured the anatomy of inner tree rings formed when the plant was in a denser stand (before harvesting, *t*_0_) with the crown concentrated in the uppermost part of the stem and the outermost rings corresponding to rings formed after harvesting when only a few trees remained and a multitude of epicormic shoots along the stem had sprouted (*t*_1_). The large sprouting of these new shoots has the effect of reducing the average hydraulic path length because the epicormic shoots are closer to the stem base (Figure 1b).

We aimed to demonstrate that this crown shape change affects the xylem structure at the stem base. Our hypothesis is that the development of epicormic shoots leads to the production of a larger number of narrower conduits hydraulically connected to these new shoots in addition to the wider conduits hydraulically connected to the uppermost leaves (Figure 1b). If many narrower conduits are produced in the outermost rings, this would indicate that hydraulic path length is the driver of conduit size. Such a result would falsify the supposed causal relationship between cambial age and conduit size, as the older meristem produced narrower, rather than wider, conduits.

## Materials and methods

### Samplings and experimental design

The study site is located in central Italy, near Macerata, in the Marche region within the Regional Nature Reserve of Monte San Vicino and Monte Canfaito (43° 19.4627′; 13° 04.6039′) at an elevation of about 900 m a.s.l. Sampling was conducted just before the end of the growing season, in late August (year 2020), when the leaves were still on the trees and the growth ring could be considered almost fully formed.

To test the hypothesis of a causal link between the sprouting of epicormic shoots and stem base xylem anatomy, we took advantage of the high crown shape plasticity of broadleaved species in response to changing light conditions (Canham 1988, Jucker et al. 2015, Kröner et al. 2024). To observe the effects of sudden changes in light availability, we exploited a silvicultural system known as coppice-with-standards, which is widely practised in southern Europe, particularly in central Italy. In this system, broadleaved woodlands are traditionally managed by cutting all trees except for ~100 individuals per hectare—the standards—which are left standing. The typical rotation period is 10–20 years. The sudden exposure to full light and increased resource availability frequently induces the sprouting of numerous epicormic shoots along the stems of these uncut (standing) trees in the years following the cut (Figure 1b).

Seven of these uncut trees (four species: *Acer opalus obtusatum* (Waldst. & Kit. Ex Willd.) Gams, *O. carpinifolia* Scop., *C. betulus* L., *Sorbus aria* (L.) Crantz.) with a substantial proportion of epicormic shoots were sampled in a stand that had been harvested 3 to 4 years prior. The number of epicormic shoots can vary between trees, so we categorized the samples into two groups based on visual assessment of epicormic shoot abundance: ‘high’ and ‘low’. The abundance was classified as ‘high’ if more than 50% of the stem below the main upper crown produced epicormic shoots and ‘low’ if less than 50% sprouted shoots. Photographs of the sampled trees and the immediate post-harvesting stand conditions are provided in Figure S1, available as Supplementary Data at *Tree Physiology* Online.

Samples have been coded using a capital letter corresponding to the genus name, followed by lowercase letters corresponding to the species name and a final subscript letter corresponding to the abundance of epicormic shoots; e.g., a *Carpinus betulus* with ‘high’ epicormic shoot abundance is Ca_H_.

For each of the following three species—*A. opalus obtusatum*, *Ostrya carpinifolia* and *Carpinus betulus*—we sampled one tree with a high and one with a low number of epicormic shoots (Table 1). Additionally, a specimen of *Sorbus aria* was included in the study because of its exceptionally high abundance of epicormic shoots, despite the absence of corresponding low-abundance samples in the area. Furthermore, we sampled two *A. opalus obtusatum* trees (Aoo1_N_, Aoo2_N_) without any epicormic shoots (categorized as ‘none’) from an unharvested stand with high stand density. Since these trees did not experience significant changes in crown vertical distribution, in line with our hypothesis, we expected no significant change in conduit size at the stem base, thereby providing a reference to further validate the primary role of hydraulic path length in influencing xylem traits. The analysis of the two samples without epicormic shoots also helped us to exclude the influence of environmental factors during the years considered for the analysis.

**Table 1:** Main characteristics of the sampled trees, showing epicormic shoot abundance, tree height and diameter at breast height (DBH). The abundance of epicormic shoots was estimated as ‘high’ (H, in sample code) if more than 50% of the stem below the pre-harvesting crown sprouted shoots; ‘low’ (L, in sample code) if less than 50% sprouted shoots; or ‘none’ (N, in sample code) in the two samples collected in the not-harvested area.

Sample code	Species	Epicormic shoots abundance	Tree height (m)	DBH (cm)
Sa_H_	*Sorbus aria*	High	6.0	5.5
Cb_H_	*Carpinius betulus*	High	9.0	12.5
Cb_L_	*Carpinus betulus*	Low	11.0	15.9
Oc_H_	*Ostrya carpinifolia*	High	14.0	27.0
Oc_L_	*Ostrya carpinifolia*	Low	13.5	16.5
Aoo_H_	*Acer opalus obtusatum*	High	11.0	11.0
Aoo_L_	*Acer opalus obtusatum*	Low	9.0	11.0
Aoo1_N_	*Acer opalus obtusatum*	None	11.0	12.0
Aoo2_N_	*Acer opalus obtusatum*	None	10.2	11.6

### Anatomical cross-section preparation

In each tree, two cores were extracted at breast height (1.30 m) using a 5 mm Pressler increment borer, with a 180° angle between them. In one case (*S. aria*—Sa_H_), due to the small dimensions of the tree (6 m height, 5.5 cm diameter), the entire plant was felled, and a complete wooden disc was collected instead of using the increment borer.

Anatomical cross-sections were prepared from the wood samples using a rotary microtome (RM2245 Leica, Heidelberg, Germany), with section thicknesses ranging from 12 to 25 μm. The preparation process involved soaking the specimens for varying lengths of time and heating them in a water and glycerol solution. In some cases, particularly with species exhibiting wider conduits (e.g., tallest individuals or ring-porous species), a non-Newtonian fluid based on cornstarch was applied (Schneider and Gärtner 2013). compared with the original formulation (primarily designed for conifers), we increased the cornstarch proportion (to achieve a more viscous solution capable of filling large conduits) and the glycerol proportion (to prevent solution solidification).

After sectioning the wood slices, tannin-rich species were bleached with sodium hypochlorite. The sections were then stained using a 1% Safranin and 0.5% Astrablue solution. Following staining, they were cleaned with water and dehydrated using ethanol in progressively increasing concentrations (50 and 100%). The sections were permanently mounted using Eukitt NEO or Eukitt UV (O. Kindler GmbH). Images were captured with a D-Sight 2.0 scanner (A. Menarini Diagnostics s.r.l., Italy) at 200× magnification.

### Analysis of conduit size and statistical analysis

Anatomical images were analysed using ROXAS software (Wegner et al. 2013, von Arx and Carrer 2014, von Arx et al. 2016, Prendin et al. 2017), which identifies and measures every vessel within the selected growth rings. To ensure accuracy, the automatic detection was checked and manually corrected by adding missing vessels and excluding xylem elements mistakenly identified as vessels.

To assess the effect of epicormic shoots sprouting in the lower portion of the stem on conduit size at the stem base, we compared rings formed after harvesting (*t*₁) with the same number of rings formed just before the year of harvesting (*t*₀), as epicormic shoots typically sprout in the year following harvesting. In samples from stands harvested 3 years before sampling (Sa_H_, Cb_H_, Oc_H_, Oc_L_), the last three rings (2018–20) were compared with the three preceding rings (2015–17). The other samples (Cb_L_, Aoo_H_, Aoo_L_) were from a stand harvested four years before sampling, so the last four rings were compared with the four preceding rings (Table 2). For the two samples without epicormic shoots from the unharvested area (Aoo1_N_, Aoo2_N_), the last four rings were compared with the four preceding rings to make the results comparable with those from trees with epicormic shoots.

**Table 2:** Main characteristics of the xylem produced in the seven trees that sprouted epicormic shoots in the lowest portion of the stem (sampled in the harvested stand). The effect of epicormic shoot sprouting was tested comparing the last three or four rings formed after harvesting (*t*_1_), and the three or four rings formed before the harvesting year (*t*_0_) when trees were still within a dense community and the crown was inserted mainly at the tree top. Mann–Whitney U test showed a significant (all *P* < 0.01) decrease in median conduit size after harvesting rings (*t*_1_), with reduction ranging from 0.93 (Cb_H_) to 0.56 (Oc_H_).

**Sample code**	**Time (pre-/post- harvesting)**	**Considered years (i.e., growing seasons pre/post harvesting)**	**Ring width sum (mm)**	**Conduit lumen area statistics**			
				**No. conduits**	**Median conduit area (μm** ^**2**^**)**	**Ratio of median conduit area (*t***_**1**_ **/*t***_**0**_ **)**	**Mann–Whitney’s U test *P*-value (*P*)**
**Sa**_**H**_	*t*_0_	−3	2.1	1974	731	0.62	<0.0001
	*t*_1_	3	4.8	5225	450		
**Cb**_**H**_	*t*_0_	−3	1.3	510	1311	0.93	<0.0001
	*t*_1_	3	12.6	6037	1215		
**Cb**_**L**_	*t*_0_	−4	8.5	2406	2047	0.76	<0.0001
	*t*_1_	4	5.9	2565	1552		
**Oc**_**H**_	*t*_0_	−3	6.4	935	1554	0.56	<0.0001
	*t*_1_	3	2.8	470	866		
**Oc**_**L**_	*t*_0_	−3	4.8	765	2049	0.85	0.0072
	*t*_1_	3	8.1	1134	1755		
**Aoo**_**H**_	*t*_0_	−4	2.0	891	1461	0.90	<0.0001
	*t*_1_	4	6.7	2534	1308		
**Aoo**_**L**_	*t*_0_	−4	2.5	1401	1761	0.82	<0.0001
	*t*_1_	4	5.7	3054	1446		

Conduit size is commonly analysed by measuring either diameter, lumen area or hydraulic diameter (Sperry et al. 1994, Tyree and Zimmermann 2002). We chose to measure lumen area instead of diameter due to the frequent elongated shape of conduits in cross-sections, particularly in species like maple or hornbeam. Moreover, lumen area was preferred over hydraulic diameter because hydraulic diameter disproportionately weights the largest conduits (which are more conductive), whereas our analysis aimed to detect differences in the abundance of small conduits, which are associated with lower leaves.

Due to the non-normal distribution of conduit size within tree rings, we compared median conduit areas rather than means. All conduits measured from pre-harvest rings (*t*₀) and post-harvest rings (*t*₁) were grouped, and the median lumen area was calculated for each group. To quantify the reduction in median lumen area after harvesting, we calculated the ratio between pre- and post-harvest medians.

The distribution of lumen areas was analysed using the cumulative distribution function (CDF), implemented in GraphPad Prism 10 (GraphPad Software, LLC). The CDF orders lumen area values from smallest to largest and calculates the cumulative probability of exceedance (*P*(*X* > *x_i_*)) that a lumen area exceeds a value *x_i_*. A steeper slope in the CDF suggests a lower probability of exceedance, indicating fewer larger cells. Since epicormic shoots are expected to reduce hydraulic path length by increasing the number of leaves in the lower stem portion, we expect the CDF for post-harvest rings to be steeper (i.e., lower than the CDF of pre-harvest years).

**Figure 2:**
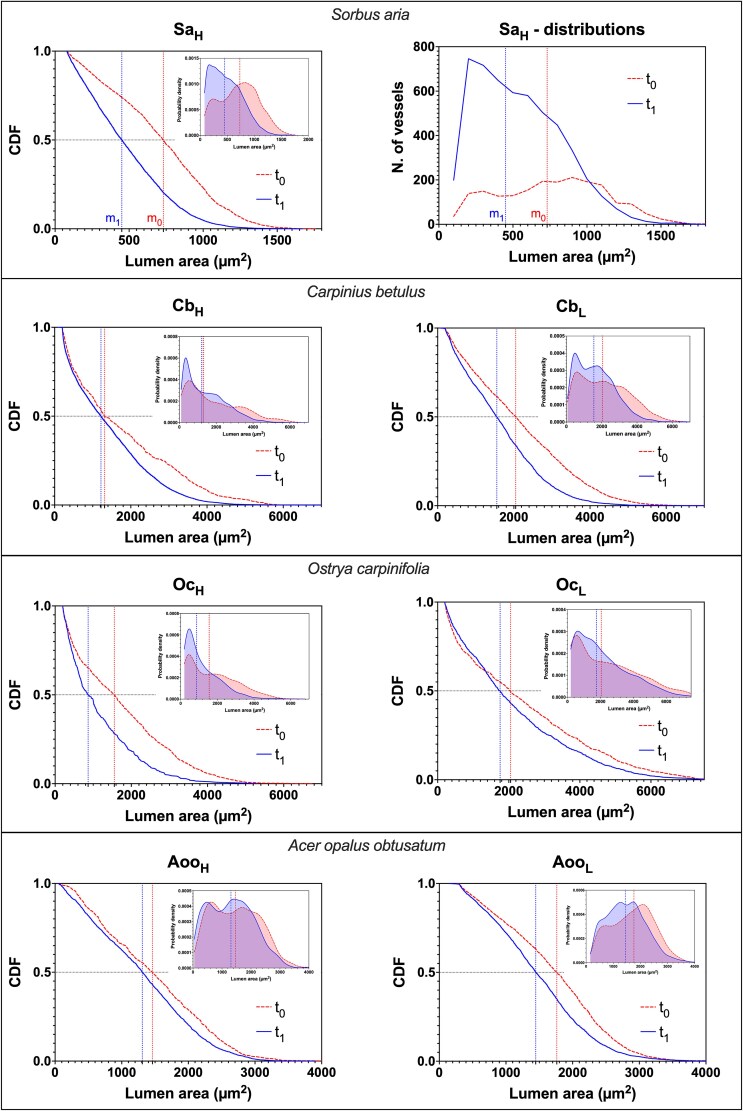
Cumulative distribution functions (CDFs) of the lumen area (μm^2^) in trees that sprouted epicormic shoots in the lowest portion of the stem (sampled in the harvested stand). Samples with a high abundance of epicormic shoots (H) are on the left, while those with a low abundance (L) are on the right, except for *Sorbus aria*, where just a ‘high’ sample is available. Solid lines (blue) represent the 3 or 4 years after harvesting (*t*_1_). Dashed lines (red) represent the same number of years but considered before harvesting (*t*_0_). A horizontal black dotted line marks the 0.5 value of the CDF (i.e., the median), while two vertical dotted lines indicate the median lumen areas before (*m*₀) and after (*m*₁) epicormic shoot sprouting, with *m*₁ always smaller (line further to the left). All samples show a significant decrease in median conduit size after the sprouting of low epicormic shoots (all *P* < 0.01). For the sample Sa_H_ frequency distributions of lumen area values are also shown (top right), highlighting the higher number of small conduits at *t*₁ and the resulting leftward shift of the distribution. It is also evident the higher number of conduits in the years at *t*_1_. Insets: Kernel density estimations (KDEs) of the lumen area distributions for *t*₀ (dashed line, red) and *t*₁ (solid line, blue). The KDEs illustrate the smoothed and normalized probability density distributions (area under each curve = 1), highlighting the left-shift toward smaller lumen areas after epicormic shoot development.

The CDF was preferred over frequency distributions because it eliminates the need for subjective class size definitions, which can affect significance, and normalises for differences in cell numbers, enabling effective comparisons between rings of different widths. Frequency distributions were also computed (using 100 μm^2^ class widths) but are presented only for Sa_H_ (Figure 2) as an example.

To test the significance of differences observed in median ratios and CDFs, we performed a nonparametric Mann–Whitney U test. This test was chosen due to the non-normal distribution of conduit sizes. The analysis was conducted using Past4 (Hammer et al. 2001).

## Results

In five of the seven trees with epicormic shoots, ring width increased following the sudden increase in light availability, whereas in two trees (Cb_L_ and Oc_H_), it decreased (Table 2). Across all samples, the change in ring width before and after harvesting was not significant (nonparametric two-sample paired comparison). In all seven samples with epicormic shoots, the median xylem conduit area significantly decreased (*P* < 0.01 for all samples) within the rings formed after the development of epicormic shoots in the lower portion of the stem (*t*₁), with reductions ranging from 0.93 to 0.56 compared with the median conduit area of rings formed before harvesting (*t*₀), when no epicormic shoots were present (Table 2). The CDFs of conduit lumen areas at *t*₁ are visibly left-shifted compared with those at *t*₀ in each sample (Figure 2).

In all samples, this increase in narrow conduits occurred simultaneously with the continued production of wider conduits, ensuring sufficient water supply to the uppermost leaves (Figure 1b).

**Figure 3:**
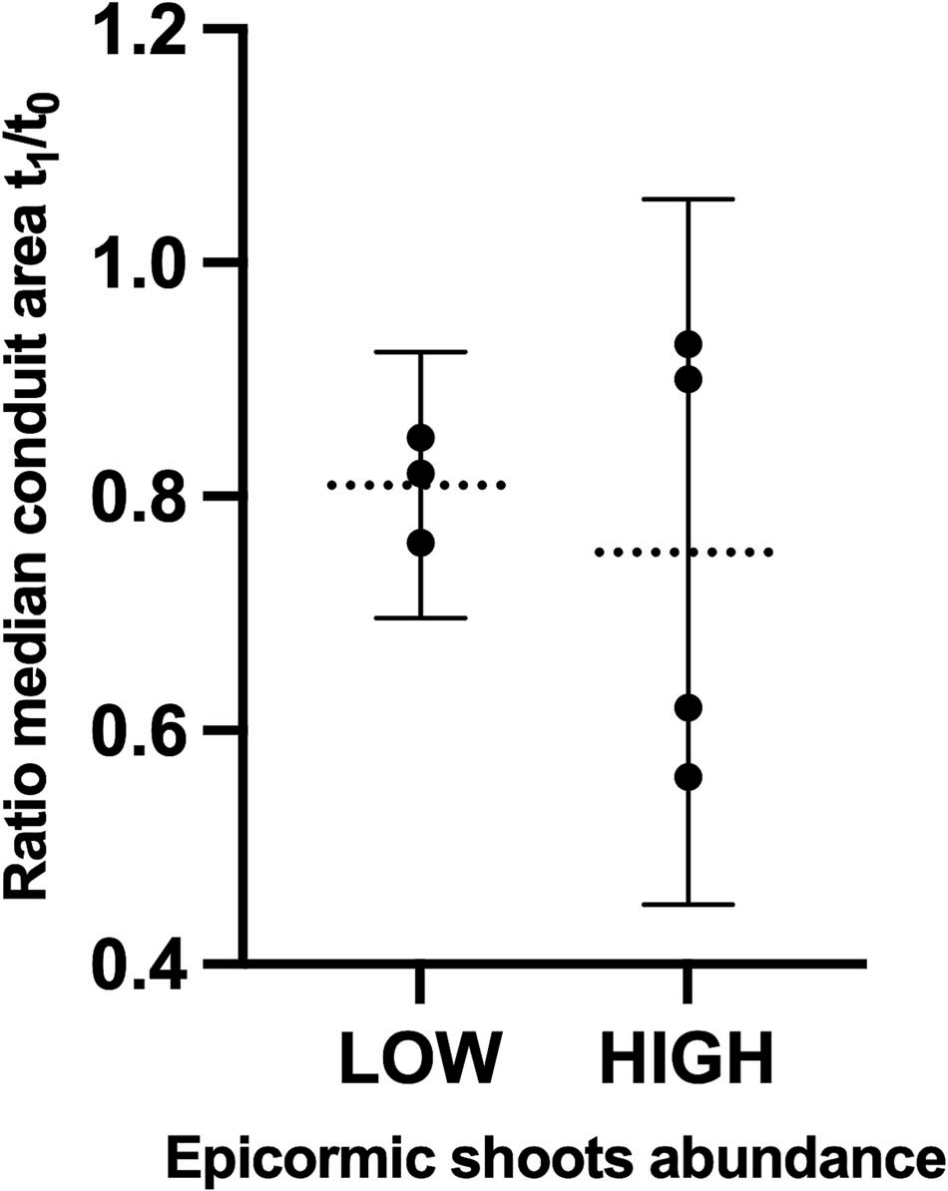
Ratio between median conduit area at *t*_1_ and *t*_0_ grouped by low or high abundance of epicormic shoots. The overlapping CIs (95% confidence intervals) suggest no significant difference between plants with low and high numbers of shoots.

There was no consistent relationship between the abundance of epicormic shoot production (high or low) and the degree of the reduction in median conduit lumen area. Trees with a greater number of epicormic shoots did not necessarily exhibit a larger decrease in median conduit lumen area. When comparing trees with high epicormic shoot production to those with low production, the difference was not significant (Table 3 and Figure 3).

**Table 3:** Descriptive statistics of the ratio between median conduit area at *t*_1_ and *t*_0_ grouped by *low* or *high* abundance of epicormic shoots. The overlapping 95% confidence intervals (CIs) suggest no significant difference between plants with low and high number of epicormic shoots.

Ratio median conduit area *t*_1_/*t*_0_	Low	High
Mean	0.81	0.75
Std deviation	0.046	0.19
Std error of mean	0.026	0.095
Lower 95% CI of mean	0.70	0.45
Upper 95% CI of mean	0.92	1.1
Coefficient of variation	5.7%	25%

Diameter at breast height (DBH) and plant height did not affect the degree of reduction in median conduit lumen area (see Figure S2 and Table S1 available as Supplementary Data at *Tree Physiology* Online).

**Figure 4:**
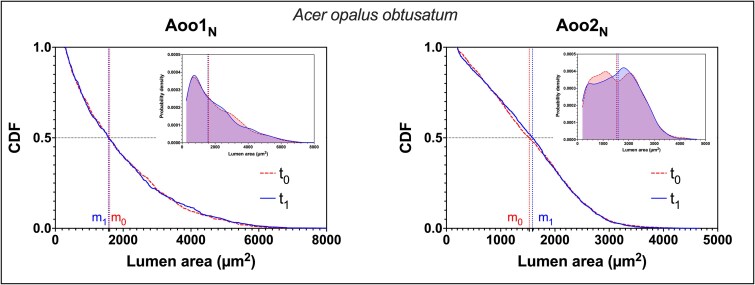
Cumulative distribution functions (CDFs) of lumen area (μm^2^) for trees sampled in the unharvested stand, where the crown was concentrated at the top and no epicormic shoots were present along the stem. Similar to the analysis of trees from the harvested stand (Figure 2 and Table 2), the last four rings (2017–20, *t*₁) were grouped and compared with the previous four rings (2013–16, *t*₀). A horizontal black dotted line marks the 0.5 value of the CDF (i.e., the median), while two vertical dotted lines indicate the median lumen areas before (*m*₀) and after (*m*₁) epicormic shoot sprouting, with lines matching the corresponding CDF (dashed, red: *t*₀; solid, blue: *t*₁). No significant differences in median conduit size were detected (all *P* > 0.01). Insets: KDEs of the lumen area distributions for *t*₀ (dashed line, red) and *t*₁ (solid line, blue). The KDEs illustrate the smoothed and normalized probability density distributions (area under each curve = 1), highlighting the lack of a left-shift between the two distributions.

In the two samples of *A. opalus obtusatum* without any epicormic shoots (Aoo1_N_ and Aoo2_N_) ring width didn’t exhibit any detectable trend over the last decade (Table 4). Upon comparison with the same group of years of the trees with epicormic shoots (analogous to pre-harvesting—*t*_0_, and post-harvesting—*t*_1_), no statistical differences were found in the values of median conduit size (Table 4). The CDFs largely overlapped, indicative of similar conduit size distributions (Figure 4).

**Table 4:** Main characteristics of the xylem produced in the two trees sampled in the unharvested area, with the crown inserted mainly at the tree top and no epicormic shoots along the stem. Similar to the analysis of trees from the harvested stand (Figure 2 and Table 2), the last four rings (2017–20, *t*₁) were grouped and compared with the previous four rings (2013–16, *t*₀). Mann–Whitney U test showed no significant difference in median conduit size (all *P* > 0.01).

**Sample code**	**Rings considered**	**Ring width sum (mm)**	**Conduit lumen area statistics**			
			**No. conduits**	**Median conduit area (μm** ^**2**^ **)**	**Ratio of median conduit area (*t***_**1**_ **/*t***_**0**_ **)**	**Mann–Whitney’s U test *P*-value (*P)***
**Aoo1**_**N**_	5–8 years old (*t*_0_)	2.2	857	1953	1.00	0.9194
	4 most recent (*t*_1_)	2.5	757	1957		
**Aoo2**_**N**_	5–8 years old (*t*_0_)	4.8	1767	1527	1.04	0.8004
	4 most recent (*t*_1_)	3.6	1366	1585		

## Discussion

### Experimental evidence of the causal relationship ‘path length–conduit size’

We based our predictions on theoretical arguments (West et al. 1999, Lechthaler et al. 2020, Koçillari et al. 2021) and empirical evidence (Anfodillo et al. 2006, Lintunen and Kalliokoski 2010, Petit et al. 2014, Petit et al. 2016, 2018, Liu et al. 2019, Olson et al. 2020, 2021) demonstrating that the hydraulic architecture of xylem is inevitably characterized by narrower conduits close to the leaves and much wider conduits towards the stem base, and that this basipetal widening is essential to counteract the increase in hydraulic resistance that would arise if conduit diameter remained constant along the entire hydraulic path.

We tested the causal link ‘path length–conduit size’ in suddenly isolated trees that sprouted many short epicormic shoots along the stem (Figure 1b). Our approach allowed us to evaluate how changes in hydraulic path length influenced conduit diameters within the same individual over time, minimizing confounding factors associated with inter-individual comparisons.

Due to the presence of numerous leaves closer to the stem base (i.e., shorter paths), we expected many narrower conduits in tree rings formed after trees were isolated, so the distribution of conduit size at the stem base should shift to the left, and the median conduit size should decrease. The results clearly supported this hypothesis, and in all measured trees, median conduit size decreased in the outermost rings (Table 2), because of the presence of many narrower conduits (Figure 2). The trees without epicormic shoots (Aoo1_N_ and Aoo2_N_), which grew in unharvested stands, showed no significant variation in conduit size (Figure 4 and Table 4). This suggests that climatic or other environmental factors did not significantly influence conduit size within trees growing under the same site conditions.

The link we observed between leaf position and conduit diameter at the stem base suggests that newly formed leaves regulate the development of the entire conduit network extending down to the roots (Aloni and Zimmermann 1983). Consequently, each leaf is supplied by a bundle of conduits, with the axial variation in diameter being primarily determined by hydraulic path length, while the number of conduits depends on the number and size of the leaves produced (i.e., leaf area). As leaf area increases due to epicormic shoots sprouting, the number of conduits in the stem also rises. Indeed, the number of vessels in rings formed after epicormic shoot development was consistently higher across samples (see Sa_H_ distributions as an example, Figure 2). A wider crown results in higher evaporative water loss, and this increased transpiration demand is met by the newly formed conduits, ensuring that the flow rate per unit of leaf area remains relatively constant.

Our results also support the hypothesis that hydraulic pathways are axially sectored. In the rings formed after harvesting, we observed the development of both narrower conduits, likely supplying the lower leaves, and wider conduits, connected to the upper canopy (Figure 1b). A higher proportion of lower leaves (as a result of the sprouting of epicormic shoots) led to a shift in the median conduit size towards narrower diameters, suggesting that the narrow vessels should be connected to those lower leaves. In scenarios where the uppermost portion of the crown undergoes partial desiccation after being abruptly exposed to light due to stand harvesting, as observed in sample Sa_H_, the post-harvesting distribution (*t*₁) shows a clear lack of the widest diameters. The right tail of the post-harvesting distribution (Figure 2) lies below that of the pre-harvesting distribution, indicating a loss of large vessels. This suggests that the widest vessels were previously (*t*₀) supplying the uppermost leaves.

This predominant axial sectoriality underpins the model of West et al. (1997), which proposes a ‘one conduit–one leaf’ relationship. Given our observations, this model appears to better represent tree hydraulic architecture than the alternative proposal of Savage et al. (2010), which suggests significant conduit coalescence towards the base, a structure that would entail less sectoriality as fewer vessels at the base supply many leaves at the apex (‘one conduit–many leaves’ structure). Reduced sectoriality would imply a weaker link between conduit size at the base of the stem and leaf height. However, our findings indicate that conduit size distribution at the base of the stem is strongly influenced by the vertical distribution of leaves.

Clear axial sectoriality has been confirmed by studies that directly assessed sap flow variation after partial leaf removal and dye movement along the stem in grapevines (McElrone et al. 2021). These studies also show that while conduits are highly interconnected, there are evident preferential pathways—those with the lowest axial resistance—which ideally correspond to the ‘tubes’ proposed by West et al. (1999). Such techniques could provide an effective way to better understand the relationship between the diameter of specific conduits and the height of the leaves they supply.

Our approach disentangled the effects of hydraulic path length and cambial age on conduit size and wood traits, challenging the widely held idea that cambial age significantly influences xylem anatomy—a notion that has already been questioned by several studies (Rosell and Olson 2007, Petit et al. 2008, Carrer et al. 2015, Cary et al. 2020, Petit 2024). The common observation that older trees have wider conduits simply overlooks the fact that older trees are typically taller and thus have longer hydraulic paths.

In actively growing trees, in fact, hydraulic path length, cambial age and organ size typically increase in parallel and are correlated with each other, making it difficult to isolate the primary physiological driver of conduit diameter. If hydraulic path length is not explicitly considered, the observed correlation between conduit size and cambial age or organ size may lead to the mistaken conclusion that age is the causal factor (Carrer et al. 2015). We do not question the results of studies reporting a correlation between cambial age and xylem conduit size; rather, we challenge the conclusion that cambial age is a causal factor, and we argue that hydraulic path length is the true determinant.

To detect the primary causal factor, we selected a particular growing condition where cambial age and hydraulic path length followed different trajectories. After a sudden increase in light availability, the sampled trees produced numerous epicormic shoots in the lower stem portion reducing hydraulic path length, while cambial age continued to increase. If cambial age dictated conduit size, we would expect wider conduits in the outer rings at the stem base formed after epicormic shoots sprouted. On the contrary, our measurements revealed a shift towards narrower conduits, supplying the newly developed leaves closer to the stem base, reinforcing hydraulic path length as the primary driver of xylem conduit diameter.

### Methodological issues and possible sources of variability

Within the forest stand, we selected individuals with evident epicormic shoots sprouting along the stem, leading to a certain ‘unbalanced’ sampling in terms of considered species. However, we believe this had minimal impact on our findings, as the tip-to-base widening of conduits is a well-conserved trait across species (Anfodillo et al. 2013, Olson et al. 2021), across diverse climates (Olson and Rosell 2013, Olson et al. 2018, 2020, 2021, Fajardo et al. 2020, Rita et al. 2024) and at all stages of tree development (Petit et al. 2023, Rita et al. 2024).

However, the current extent of our measurements does not provide the necessary detail to formulate a precise, comprehensive model. The abundance of epicormic sprout on a tree does not always correspond to the relative difference between pre- and post-harvesting conduit sizes. A tree with a high number of epicormic shoots may not necessarily exhibit a greater difference in conduit size compared with a tree with a modest shoot sprouting (Figure 3). For example, the species *C. betulus*, sample Cb_L_—characterized by low sprouting of epicormic shoots—exhibited a more substantial reduction in median conduit diameter (*t*_1_/*t*_0_ = 0.76), compared with sample Cb_H_, which, despite high shoot sprouting, showed a small reduction in median conduit size (*t*_1_/*t*_0_ = 0.93). The most plausible interpretation of these observations is that after harvesting, higher resource availability leads to a generalized growth across the entire tree crown, not just the sprout of low epicormic shoots. The upper portion of the crown experienced significant enlargement, leading to the production of larger vessels at the stem base and shifting the distribution to the right (increasing the median). This top-crown growth may partially mask the leftward shift of the distribution (decreasing of the median) caused by the numerous smaller vessels associated with the lower epicormic shoots. It is therefore reasonable to assume that trees producing more epicormic shoots also expanded their upper crown more, generating larger vessels at the stem base and balancing the increased number of smaller ones. More precise predictions on changes in conduit size could be made if variation in crown shape within a single tree were measured, e.g., using a terrestrial laser scanner (Sheppard et al. 2017, Kröner et al. 2024), which, probably, is the only available technology to accurately measure the length of all hydraulic paths associated with a post-harvesting crown growth.

Another potential source of variability may arise from the fact that new shoots often bear leaves that are longer than those found in the rest of the crown. Since in broadleaf species the rate of conduit widening within leaves is very high (Lechthaler et al. 2019, Olson et al. 2021), very wide conduits are found at the base of the petiole in long leaves. This means that large conduits are also present at the tip of the twig supporting them and, consequently, wider conduits are found in the stem as well. For this reason, it would always be important to measure leaf length and the rate of widening along the midrib and petiole, as the hydraulic pathway effectively ‘begins’ near the stomata.

A further measurement complexity arises from the inherent anatomical structure of the tree, in which conduits progressively widen with increasing distance from the apex, following approximately a power law pattern with an exponent of about 0.2 (Anfodillo et al. 2013, Olson et al. 2021). This implies that the greatest absolute variation in conduit diameter occurs within the uppermost portion of the stem (i.e., 0 to 2–3 m below the tip). If epicormic shoots develop not only near the base but also along the mid-height portion of the stem, the conduit size associated with these mid-height leaves may differ only slightly from those supplying the upper canopy. As a result, the overall variation in conduit size distribution after epicormic shoot formation may be limited.

In scenarios where the uppermost portion of the tree crown undergoes partial desiccation—a phenomenon potentially observed when a tree is abruptly freed from dense cover—the average length of the hydraulic pathways markedly decreases, leading to a clear leftward shift in the distribution of conduit sizes. This situation represents an optimal condition to test our hypothesis and was observed in sample Sa_H_, where at *t*_1_ there is a clear lack of the widest diameters, which at *t*_0_ were supplying the uppermost leaves (Figure 2).

The magnitude of the response we observed may also depend on the size of the leaves produced by epicormic shoots. Conduit basipetal widening is particularly steep in broadleaved trees, with reported scaling exponents above 0.4 (Coomes et al. 2008, Lechthaler et al. 2019, Levionnois et al. 2020), so leaf size strongly influences conduit diameter along the entire underlying hydraulic path. It is therefore possible that epicormic branches produced larger leaves than those in the upper crown, leading to the formation of larger vessels at the stem base than would be expected if leaves were of equal size throughout the crown. However, we did not measure leaf size along the plants, so this remains a hypothesis that future work should test.

### Insights into modelling xylem conduit size

The complexity of the conductive network—comprising a vast number of interconnected cells that form multiple hydraulic pathways supplying leaves at varying distances—makes modelling its architecture particularly challenging. However, anatomical traits are known to be highly correlated (e.g., pit permeable area scales isometrically with conduit area; Lazzarin et al. 2016), suggesting that considering a single trait may implicitly account for others. Koçillari et al. (2021) developed a model to predict the shape of the entire hydraulic path of a single conduit element from root to leaf. By considering conduit diameter as the primary variable determining flow rate and applying two optimization principles—maximizing water conductance while minimizing carbon costs—the model generates theoretical profiles that closely match those empirically observed in nature. This suggests that while other key anatomical traits (e.g., pits and perforation plates) are crucial for hydraulic transport and embolism resistance (Carlquist 2001, 2018, Lens et al. 2011, Bouche et al. 2014, Medeiros et al. 2019, Zambonini et al. 2024), they likely vary in parallel with conduit diameter (Sperry et al. 2006, Schulte 2012, Lazzarin et al. 2016) to maintain xylem functionality. Consequently, modelling xylem architecture can be effectively simplified by focusing primarily on conduit diameter.

The results can also be used to better understand and model the role of turgor in determining xylem conduit size. It was observed that with higher water availability, cambium tends to form wider xylem conduit cells (Cabon et al. 2020) but it has also been reported that the cells produced during controlled water deficit periods were not significantly narrower (Petit et al. 2016, 2022, Kiorapostolou et al. 2018, Petit 2024), especially when the necessary adjustment for distance from the apex has been applied (Olson et al. 2020, Petit et al. 2022, Petit 2024). However, water deficit often led to a reduction in the number of cells produced as cambial activity decreases under such conditions (Deslauriers et al. 2016). Our data suggested a relatively weak link between cell size and water availability. After partial harvesting, resource availability increases for the few remaining trees, leading to an overall increase in the number of cells produced and thus ring width (in five samples out of seven). Yet, in our case, increased water availability was also associated with a reduction in median conduit size, supporting the notion that, even with greater water availability, xylem conduit size is primarily regulated in relation to the distance from the leaves being supplied rather than by turgor-driven limitations (Segovia-Rivas and Olson 2023). More resources can lead to the production of more conduits (wider rings) in parallel with the increase of leaf area, but the diameter of these conduits is strictly determined by the distance from the leaves (i.e., hydraulic path length).

The exact physiological mechanism through which trees regulate xylem conduit size remains to be elucidated. The polar transport of auxin from leaves through the vascular cambium is considered an important mechanism linking leaf development to xylem development (Aloni and Zimmermann 1983) but its precise role in determining vessel diameter remains unclear, especially in very tall trees (Hacke et al. 2017).

## Conclusions

Our experiment highlights a potentially valuable approach to investigating the influence of hydraulic path length on xylem conduit size in branches and stems. The observed changes in crown structure and shape predictably affect conduit size at the stem base, supporting the idea of a causal relationship between leaf position and xylem anatomy. These findings provide evidence that challenges the notion of a direct causal link between cambial age and conduit size.

Moreover, our results suggest that hydraulic pathways may be largely axially sectored, with leaves at different heights being supplied by distinct conduit bundles. This implies that, although xylem forms an anatomically interconnected network, water transport within a tree may occur mainly through hydraulically independent pathways.

Our study once again demonstrates the essential need to take the length of the hydraulic pathway into account when measuring and interpreting variations in xylem anatomical traits.

## Supplementary Material

Bicego_et_al_SD_Figure_S1_tpaf127

Bicego_et_al_SD_Figure_S2_Table_S1_tpaf127

Bicego_et_al_SD_tpaf127

## Data Availability

The raw data (all anatomical measurements, more than 50,000 cells) underlying this article will be shared upon reasonable request to the corresponding author.
