# Supplementary material for: Hydraulic path length as a determinant of xylem conduit size at the stem base, regardless of cambial age
Source: Tree Physiol. 2025 Oct 14;45(11):tpaf127. doi: 10.1093/treephys/tpaf127 (PMC12636518; doi:10.1093/treephys/tpaf127)
Supplement: Bicego_et_al_SD_Figure_S1_tpaf127 [file bicego_et_al_sd_figure_s1_tpaf127.docx]

**SI**

| **Aoo_H_** | **Cb_H_** | **Oc_H_** |  |
| --- | --- | --- | --- |
| 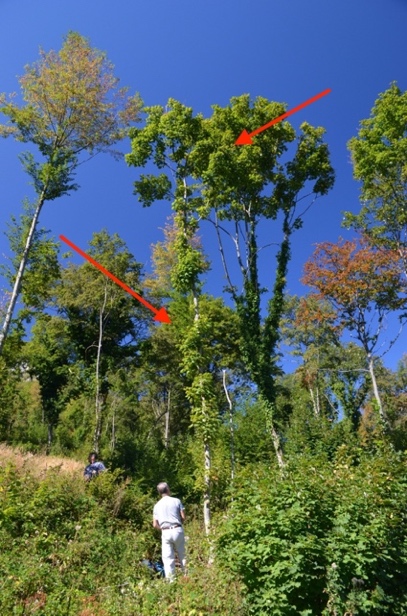 | 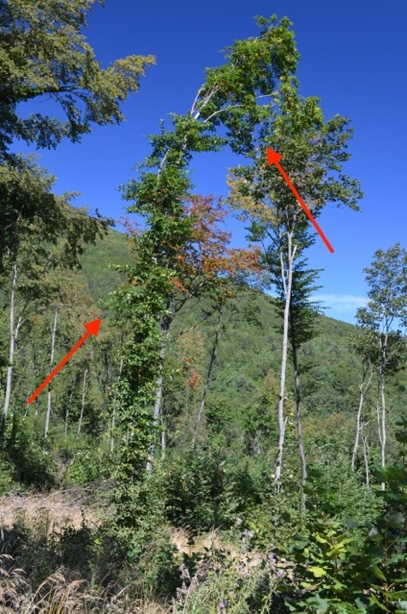 | 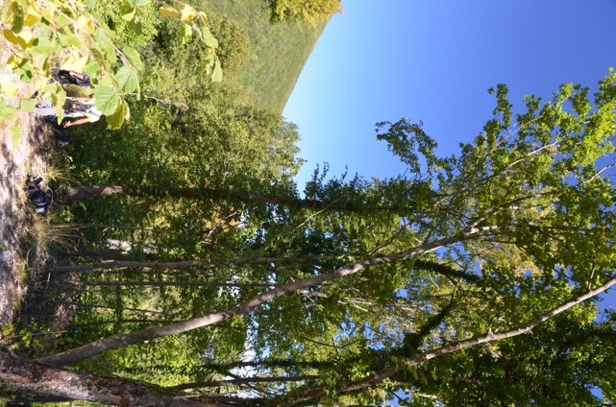 |  |
| **Aoo_L_** | **Cb_L_** | **Oc_L_** |  |
| 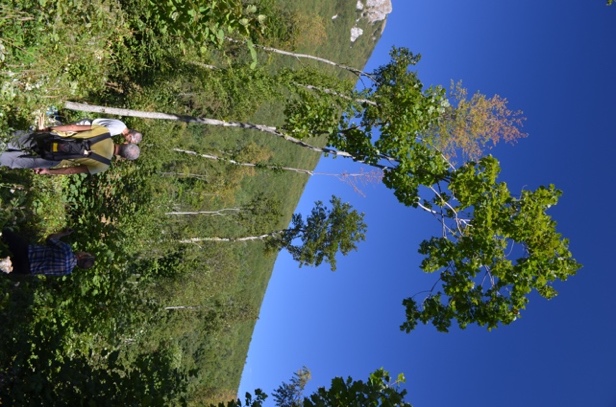 | 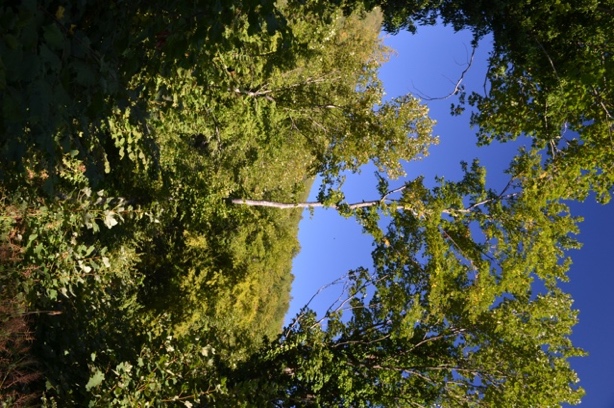 | 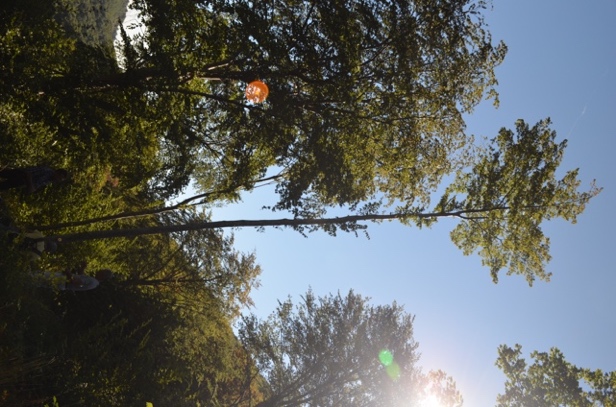 |  |
| **Sa_H_** | **Example of *coppice-with-standards* technique**  (1 year after the coppicing cut) | | |
| 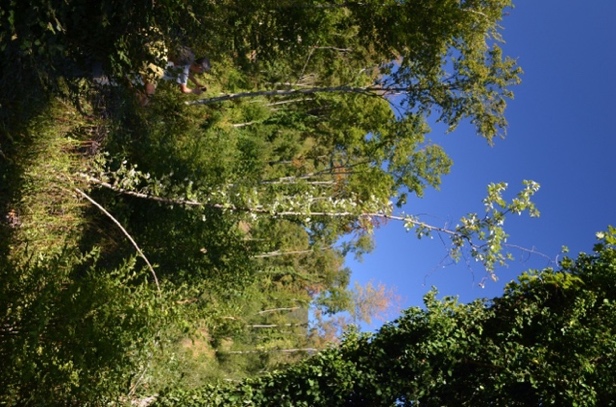 | 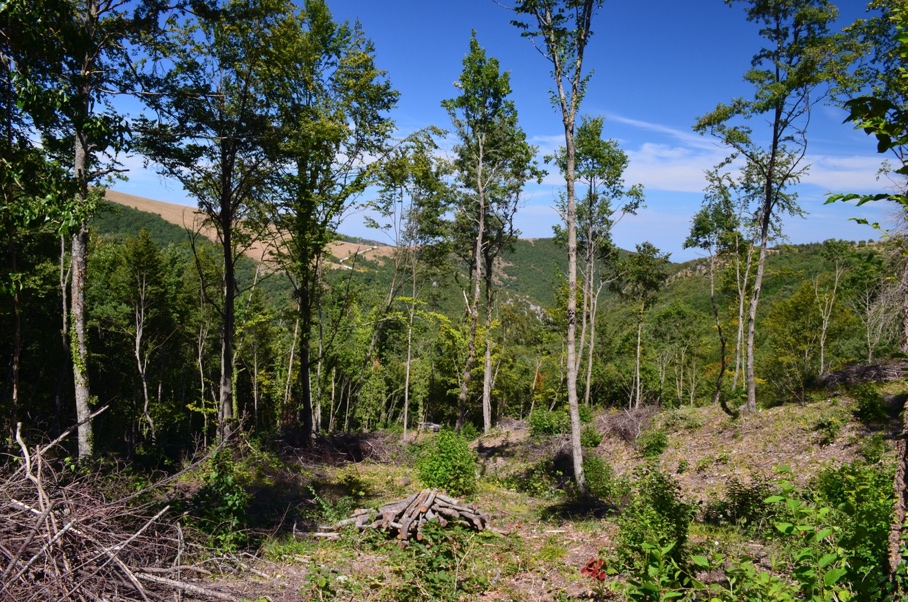 | | |

**FIG. S1**

**Figure S1** – Photographs of the seven trees that developed epicormic shoots in the lower portion of the stem (sampled in the harvested stand). Sampling was conducted 3 to 4 years after harvesting. An additional photograph of a recently harvested stand illustrates the conditions immediately following the coppicing cut, with isolated standing trees that have not yet produced epicormic shoots.
