# Supplementary material for: Hydraulic path length as a determinant of xylem conduit size at the stem base, regardless of cambial age
Source: Tree Physiol. 2025 Oct 14;45(11):tpaf127. doi: 10.1093/treephys/tpaf127 (PMC12636518; doi:10.1093/treephys/tpaf127)
Supplement: Bicego_et_al_SD_Figure_S2_Table_S1_tpaf127 [file bicego_et_al_sd_figure_s2_table_s1_tpaf127.docx]

**SI**

***Influence of plant height and diameter on conduit size reduction***

To assess whether tree size influences the reduction in median conduit lumen area following epicormic shoot sprouting in the lower portion of the stem, we examined the relationships between diameter at breast height (DBH) and tree height (H) with median lumen area before (t₀) and after (t₁) harvesting (Fig. 2 SI).

The results indicate that the slopes of the regression lines for both DBH and height remain nearly identical between t₀ and t₁, meaning that the reduction in conduit size does not systematically vary with tree size. If the slope at t₁ were higher than at t₀, this would suggest that trees with greater DBH or height experienced a smaller reduction in median lumen area. Conversely, a lower slope at t₁ would indicate that smaller trees exhibited a less pronounced reduction. However, since the slopes remain unchanged, DBH and height have no detectable influence on the degree of conduit size reduction.

The similarity in slopes and confidence intervals values (Tab. S1) suggests that changes in median conduit area are independent of DBH and height.

These findings provide further support that only the position of the leaves on the stem (hydraulic path length) and their abundance drive the reduction in median conduit size at the stem base. Neither DBH nor height affects the degree of conduit size reduction, reinforcing the hypothesis that hydraulic architecture is primarily governed by the spatial distribution of leaves.


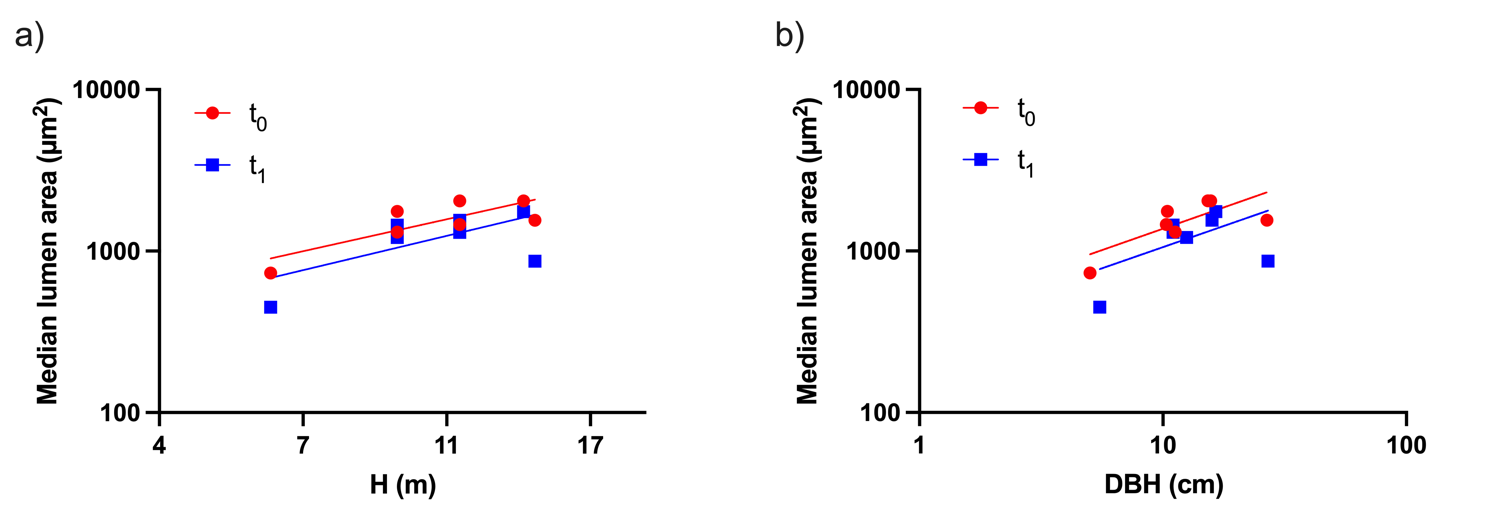


**Fig. S2** – All axes are logarithmic (log₁₀ scale). (a) Median lumen area of xylem vessels plotted against plant height (H) for both pre-harvest (t₀, red dots) and post-harvest (t₁, blue squares) rings, fitted with a power law. Plant height was measured at t₁ and assumed unchanged for t₀, given that only 3 to 4 years elapsed between t₀ and t₁, during which height growth was considered negligible. (b) Median lumen area of xylem vessels plotted against stem diameter at breast height (DBH) for both pre-harvest (t₀, red dots) and post-harvest (t₁, blue squares) rings, fitted with a power law. DBH was measured at t₁, and the cumulative width of t₁ rings was subtracted to estimate DBH at t₀.

|  | H | | DBH | |
| --- | --- | --- | --- | --- |
|  | t_0_ | t_1_ | t_0_ | t_1_ |
| Y intercept | 2.185 | 2.007 | 2.610 | 2.500 |
| Slope | 0.9895 | 1.062 | 0.5283 | 0.5240 |
| 95% CI (profile likelihood) |  |  |  |  |
| Y intercept | 1.252 to 3.460 | 0.4617 to 4.228 | 1.882 to 3.516 | 1.122 to 4.574 |
| Slope | -0.1923 to 1.966 | -0.9445 to 2.725 | -0.2111 to 1.262 | -1.079 to 1.918 |
| R^2^ | 0.6940 | 0.5763 | 0.6381 | 0.4524 |

**Tab. S1** – Summary of regression analyses examining the relationship between median lumen area of xylem conduits and plant height (H) and diameter at breast height (DBH) before (t₀) and after (t₁) harvesting. The table reports the Y intercept, slope, and R² values for the power law fits. 95% confidence intervals (CI, profile likelihood) are provided for both intercepts and slopes. The CI of the slope overlap in both cases, indicating that neither H nor DBH significantly effects the magnitude of median conduit lumen area reduction (difference between t_0_ and t_1_).
